# Integrating Molecular and Imaging Insights: Ryanodine Receptor 2 (RyR2) Expression, CK18 Status, and Conventional MRI Findings in Pituitary Neuroendocrine Tumors

**DOI:** 10.3390/ijms27188146

**Published:** 2026-09-12

**Authors:** Monika Duseikaite-Vidike, Alvita Vilkeviciute-Petraite, Indre Zostautiene, Viktorija Kasetaite, Aiste Urbonaite, Balys Remigijus Zaliunas, Lina Poskiene, Jurgita Makstiene, Sheng-Nan Wu, Vita Rovite, Ilona Mandrika, Arimantas Tamasauskas, Rasa Liutkeviciene

**Affiliations:** 1 Laboratory of Ophthalmology, Neuroscience Institute, Lithuanian University of Health Sciences, Eivenių Str. 2, LT-50161 Kaunas, Lithuania; monika.duseikaite-vidike@lsmu.lt (M.D.-V.);; 2 Radiology Department, Medicine Academy, Lithuanian University of Health Sciences, Eivenių Str. 2, LT-50161 Kaunas, Lithuania; 3 Medical Academy, Lithuanian University of Health Sciences, Eivenių Str. 2, LT-50161 Kaunas, Lithuania; 4 Department of Pathology, Medical Academy, Lithuanian University of Health Sciences, LT-44307 Kaunas, Lithuania; 5 Department of Research and Education, An Nan Hospital, China Medical University, Tainan 709, Taiwan; 6 Department of Molecular and Functional Genomics, Latvian Biomedical Research and Study Centre, LV-1067 Riga, Latvia; 7 Department of Neurosurgery, Hospital of Lithuanian University of Health Sciences, Kaunas Clinics, LT-50161 Kaunas, Lithuania

**Keywords:** pituitary neuroendocrine tumors, MRI, ryanodine receptors 2, cytokeratin 18

## Abstract

Ryanodine receptor 2 (RyR2) has been increasingly recognized as an important regulator in cancer biology. Previous studies demonstrate that RyR2 expression is associated with patient survival, although its precise role in tumorigenesis remains unclear. Elevated RyR2 expression has been linked to poorer survival outcomes, while gene silencing or pharmacological inhibition (e.g., with S107) has been shown to reduce cancer cell metastasis in both in vitro and in vivo models. Cytokeratin 18 (CK18), a key epithelial marker, is associated with tumor differentiation, hormonal phenotype, and clinical behavior in pituitary neuroendocrine tumors (PitNETs), reflecting underlying cytoskeletal organization. Variations in CK18 expression have been linked to distinct adenoma subtypes and may provide insight into tumor aggressiveness. This exploratory study investigated associations between RyR2 expression, CK18 status, and conventional MRI findings of pituitary tumors. Quantitative T2-weighted signal intensity and radiomic features were not assessed. This exploratory observational study used prospective patient recruitment between January 2017 and January 2026, after ethics approval was obtained in December 2016. RyR2 expression in immunohistological samples was quantified using QuPath v0.6.0. CK18 immunostaining was performed with the Dako Omnis system according to the manufacturer’s protocols. Conventional MRI findings were available for 24 patients and were retrospectively reviewed. Statistical analysis was conducted using SPSS/W 31.0. No statistically significant associations were observed between RyR2 expression and sex, hormonal activity, tumor size, invasiveness, recurrence, or the evaluated conventional MRI findings (all *p* > 0.05). The only statistically significant finding was higher RyR2 expression in CK18-negative than in CK18-positive tumors (median (IQR): 2.49% (18.36%) vs. 0.06% (0.39%); *p* = 0.006; r = 0.47). Higher RyR2 expression was observed in CK18-negative tumors. Given the exploratory design and limited sample size, this association should be considered hypothesis-generating and requires validation in larger independent cohorts.

## 1. Introduction

Pituitary neuroendocrine tumors (PitNETs) represent approximately 10–25% of all intracranial tumors and are detected in up to 10% of the general population [1]. Epidemiological data indicate that both their incidence and prevalence have been gradually increasing over recent decades [2]. Although PitNETs are histologically benign, a subset may display clinically aggressive behavior, presenting significant therapeutic challenges [3].

PitNETs are deemed nonfunctional when hormone secretion is insufficient to elevate serum levels or elicit clinical symptoms [4]. Prolactinomas constitute the largest subgroup (40–57%), followed by nonfunctional adenomas (28–37%), growth hormone–secreting tumors (11–13%), and ACTH-producing lesions (1–2%). Adenomas secreting FSH, LH, or TSH are rare. Tumors are also classified by size: microadenomas (<10 mm) are slightly more common than macroadenomas (≥10 mm) (57.4% vs. 42.6%) [4,5]. Macroadenomas, accounting for roughly 6–10% of all pituitary tumors, are typically clinically nonfunctional and more frequent in men. Symptoms usually stem from compression of nearby structures or from hypopituitarism [6]. Overall, clinical manifestations depend on the functional type, size, and invasiveness of the tumor [7].

Ryanodine receptor 2 (RyR2), also known as the cardiac ryanodine receptor, is a key regulator of intracellular Ca^2+^ release in cardiac myocytes [8]. Previous studies have shown that RyR2 splice variants found in the heart increase susceptibility to apoptosis [9]. Therefore, the RyR2 protein is considered one of the main regulators of cell homeostasis. Localized to the endoplasmic/sarcoplasmic reticulum, RyR2 mediates excitation–contraction coupling, the physiological process linking electrical depolarization to muscle contraction [10]. Proper RyR2 regulation is essential for normal cardiac performance, as its dysregulation has been linked to arrhythmogenic disorders and cardiomyopathies [11,12]. Beyond its role in cardiac physiology, Ca^2+^ signaling is fundamental to diverse cellular processes, and its disturbance can drive tumor cell proliferation, survival, and metastasis [13]. Aberrant RyR2 expression or regulation has been associated with multiple malignancies, including non-small cell lung, colorectal, esophageal, and breast cancers [14,15,16,17]. In particular, somatic mutations and promoter methylation of RyR2 have been implicated in the pathogenesis of head and neck squamous cell carcinoma by disrupting normal calcium homeostasis and promoting oncogenic progression [18]. Despite increasing evidence linking RyR2 to tumor biology in other malignancies, its expression and clinicopathological relevance in pituitary adenomas remain poorly characterized. This knowledge gap provided the rationale for investigating RyR2 expression in relation to histopathological and conventional MRI findings of PitNETs.

Cytokeratin 18 (CK18), an intermediate filament protein co-expressed with CK8, plays roles beyond cytoskeletal integrity, including regulation of apoptosis, cell cycle progression, and cell signaling, and its altered expression has been associated with tumor aggressiveness and prognosis across multiple cancer types [19,20]. In pituitary neuroendocrine tumors, CK18 immunohistochemistry is an established tool for histopathological classification, characteristically highlighting fibrous bodies in sparsely granulated somatotroph tumors and Crooke’s cell changes in corticotroph adenomas [21].

Magnetic resonance imaging (MRI) remains the gold standard for the diagnostic evaluation of PitNETs. It is essential not only for the initial detection and anatomical delineation of the lesion but also for longitudinal assessment of tumor progression or recurrence. The principal advantages of MRI include its high spatial resolution, multiplanar imaging capability, and excellent soft-tissue contrast. Nevertheless, conventional MRI interpretation is largely qualitative and relies heavily on the radiologist’s expertise, which introduces inter-observer variability and the risk of overlooking subtle pathological features. This inherent subjectivity has prompted the pursuit of more quantitative and reproducible image-analysis approaches [22].

This exploratory study aimed to investigate associations between RyR2 expression, CK18 status, and conventional MRI-defined tumor size, extension, and invasiveness. Quantitative T2-weighted signal intensity and radiomic features were not assessed.

## 2. Results

### 2.1. RyR2 Results

The study included 35 patients diagnosed with PitNET. The study group consisted of 15 men (42.9%) and 20 women (57.1%); the gender distribution did not differ significantly (*p* = 0.420). Based on tumor size, macroadenomas were identified more frequently than microadenomas, in 22 cases (62.9%) and 13 cases (37.1%), respectively. Tumor recurrence was observed in 16 patients (45.7%), whereas 19 patients (54.3%) showed no recurrence. Regarding tumor invasiveness, 21 tumors (60.0%) were classified as invasive, and 14 (40.0%) as noninvasive. The majority of tumors were hormonally inactive, 23 cases (65.7%), while hormonally active adenomas accounted for 12 cases (34.3%).

The associations between the percentage expression of RyR2 and various clinical and pathological characteristics of PitNETs were analyzed. RyR2 percentages are presented as median values with interquartile ranges (IQR). The Mann–Whitney U test was applied to compare groups. When evaluating tumor size, the median RyR2 percentage in the microadenoma group was 0.17% (IQR 1.79%), and in the macroadenoma group 0.105% (IQR 1.26%); the difference was not statistically significant (*p* = 0.601; effect size r = 0.09). Regarding tumor invasiveness, the median RyR2 percentage in noninvasive adenomas was 0.195% (IQR 2.73%), compared with 0.08% (IQR 0.85%) in invasive adenomas, with no statistically significant difference (*p* = 0.396; r = 0.15). By hormonal activity, the median RyR2 percentage was higher in hormonally inactive adenomas, 0.42% (IQR 2.48%), compared to hormonally active adenomas, 0.07% (IQR 0.23%); however, the difference did not reach statistical significance (*p* = 0.107; r = 0.27). In the analysis of tumor recurrence, patients without recurrence showed a median RyR2 percentage of 0.22% (IQR 3.49%), whereas those with recurrence had 0.07% (IQR 0.86%); the difference was not statistically significant (*p* = 0.230; r = 0.21). Sex-based analysis revealed that the median RyR2 percentage was 0.06% (IQR 0.36%) in women and 0.49% (IQR 2.45%) in men. The median RyR2 percentage was numerically higher in men than in women; however, the difference was not statistically significant (*p* = 0.069; r = 0.31).

The associations between RyR2 percentage values and immunohistochemical PitNET markers were evaluated. Among the 33 patients with available CK18 data, 20 (60.6%) had non-functioning PitNETs, 7 (21.2%) had prolactinomas, 4 (12.1%) had acromegaly/GH-secreting PitNETs, 1 (3.0%) had Cushing syndrome, and 1 (3.0%) had a corticotroph tumor. Analysis of CK18 status in 33 PitNET patients revealed a statistically significant difference. In the group of CK18-negative tumors, the median percentage of RyR2 was statistically significantly higher compared with CK18-positive tumors (median (IQR): 2.49% (18.36%) vs. 0.06% (0.39%), *p* = 0.006; effect size r = 0.47) (Table 1).

### 2.2. MRI and RyR2 Results

Suprasellar extension was observed in the majority of patients with PitNET. Out of 24 analyzed cases, 20 patients (83.3%) demonstrated suprasellar extension, while 4 patients (16.7%) did not. The median RyR2 percentage was lower in patients with suprasellar extension compared to those without extension (0.065% vs. 0.26%); however, this difference was not statistically significant (*p* = 0.737; r = 0.08). Among the 24 analyzed cases, 22 patients (91.7%) demonstrated parasellar extension, while only 2 patients (8.3%) showed no extension. The median RyR2 percentage was lower in patients with parasellar extension compared to those without extension (0.075% vs. 0.13%); however, this difference was not statistically significant (*p* = 0.725; r = 0.09).

Regarding the parasellar and intracranial tumor extension, minimal extension (grades 0–1) was observed in 11 patients (45.8%), while significant extension (grades 2–3) was present in 13 patients (54.2%). Median RyR2 percentages were 0.22% (IQR 0.45%) in patients with minimal extension and 0.06% (IQR 1.43%) in those with significant extension. No statistically significant difference was observed between the groups (*p* = 0.955; r = 0.02).

Paracavernous extension was identified in all 24 patients. According to the Knosp classification, true cavernous sinus invasion (grades 3–4) was present in 13 patients (54.2%), while 11 patients (45.8%) had non-invasive tumors (grades 0–2). The median RyR2 percentage was lower in patients with invasive paracavernous extension compared to those with non-invasive extension (0.06% vs. 0.22%); however, this difference did not reach statistical significance (*p* = 0.459; r = 0.16).

The inferior parasellar region is defined as tumor extension through the inferior wall of the sella turcica. Inferior parasellar extension was analyzed in 24 patients. According to the Knosp classification, true cavernous sinus invasion (grades 3–4) was identified in 14 patients (58.3%), while 10 patients (41.7%) had non-invasive extension (grades 0–2). The median RyR2 value was lower in patients with invasive inferior parasellar extension compared to those with non-invasive extension (0.055% vs. 0.125%); however, this difference was not statistically significant (*p* = 0.371; r = 0.19) (Table 2).

The association between CK18 status and MRI findings was analyzed in patients with PitNET (*n* = 23). Suprasellar extension was observed in 6 CK18-negative tumors (85.7%) and 13 CK18-positive tumors (81.2%), with no statistically significant difference between groups (*p* = 1). Parasellar extension was present in all CK18-negative cases (100%) and in 14 CK18-positive cases (87.5%), with no statistically significant difference (*p* = 1). In parasellar and intracranial extension, minimal extension was identified in 2 CK18-negative tumors (28.6%) and 8 CK18-positive tumors (50%), whereas significant extension was observed in 5 CK18-negative tumors (71.4%) and 8 CK18-positive tumors (50%). No significant association was found between CK18 expression and parasellar and intracranial extension (*p* = 0.405). According to the Knosp classification, non-invasive paracavernous extension was observed in 2 CK18-negative (28.6%) and 8 CK18-positive tumors (50%), while invasive extension was present in 5 CK18-negative (71.4%) and 8 CK18-positive tumors (50%), with no statistically significant difference between groups (*p* = 0.405). Similarly, non-invasive inferior parasellar extension was found in 4 CK18-negative (57.1%) and 6 CK18-positive tumors (37.5%), whereas invasive extension occurred in 3 CK18-negative (42.9%) and 10 CK18-positive tumors (62.5%), showing no significant association with CK18 expression (*p* = 0.650). Overall, CK18 expression was not statistically significantly associated with suprasellar, parasellar, paracavernous, or inferior parasellar MRI findings in PitNET patients (Table 2).

## 3. Discussion

RyR2 is an intracellular Ca^2+^ release channel involved in the regulation of endoplasmic/sarcoplasmic reticulum calcium homeostasis [23]. More broadly, intracellular Ca^2+^ signaling is closely linked to organelle communication and endoplasmic reticulum function [24,25], and tight regulation of intracellular Ca^2+^ concentrations is essential for numerous cellular processes [26]. Dysregulation of calcium-permeable channels and Ca^2+^ signaling has been implicated in autophagy, proliferation, and cancer progression [27,28], whereas severe or persistent endoplasmic reticulum stress can induce cell death pathways [29]. Intracellular Ca^2+^ release channels, including RyRs, have broad physiological roles [30], and experimental studies have linked RyR-dependent Ca^2+^ signaling to proliferative and immune-cell responses [31,32]. In cancer, the clinical relevance of RyR expression appears to be context-dependent: RyR expression has been inversely associated with tumor grade in breast cancer [33], whereas low RyR2 expression has been associated with adverse clinicopathological characteristics and poorer prognosis in thyroid carcinoma [34]. More broadly, altered Ca^2+^ signaling has been increasingly recognized as a potential therapeutic target in cancer [35].

Despite established connections between RyR2 and melanoma [36], breast cancer [37], prostate cancer [38], and lymphoma [39], Nizhen Xu et al. revealed that RyR2 is downregulated in thyroid carcinoma, and that low expression of RyR2 is associated with poor prognosis in patients with thyroid carcinoma. RyR2 may therefore serve as a promising tumor suppressor gene in thyroid carcinoma [34]. RyR2 could be related to the occurrence and prognosis of various cancers. In particular, high expression of RyR2 in colorectal cancer patients has been found to result in shorter survival times [15]. Compared with patients with low RyR2 expression, OSCC patients with high RyR2 expression exhibited significantly shorter overall survival [40].

Previous work by Zaliūnas et al. demonstrated a significant association between CK18 immunohistochemical patterns and clinically relevant features of PitNETs. In their study, invasive PitNETs were significantly more often characterized by negative CK18 reactions compared with non-invasive tumors (44.4% vs. 13.3%, *p* < 0.001). In addition, hormonally inactive PitNETs showed a higher prevalence of CK18 negativity compared with functionally active tumors (42.0% vs. 18.4%, *p* = 0.011). No statistically significant differences in CK18 expression were observed when comparing microadenomas with macroadenomas or tumors with and without recurrence, suggesting that CK18 loss is more closely linked to invasive growth and functional dedifferentiation rather than tumor size or recurrence alone [41].

Beyond PitNETs, CK18 has been extensively studied in a variety of malignancies, where its expression is closely related to epithelial differentiation, cytoskeletal organization, and tumor behavior. In breast cancer, elevated serum CK18 levels have been associated with poorer prognosis, whereas higher tissue expression has been linked to more differentiated tumor phenotypes and improved outcomes [42]. Conversely, suppression of CK18 expression has been observed in advanced and metastatic breast tumors, where CK18 loss promotes activation of the NF-κB/Snail signaling axis and contributes to drug resistance mechanisms, including upregulation of breast cancer resistance protein (BCRP) [43].

In other tumor types, CK18 expression appears to increase with aggressiveness. Safadi et al. reported that stronger CK18 immunohistochemical staining was significantly associated with advanced clinical stage, deeper invasion, and increased recurrence risk in oral and oropharyngeal squamous cell carcinoma [44]. In contrast, prostate cancer studies have demonstrated an inverse relationship between CK18 expression and tumor grade, with lower CK18 staining intensity observed in tumors with higher Gleason scores, suggesting CK18 downregulation in more aggressive disease [45]. These divergent findings highlight the tissue-specific and context-dependent role of CK18 in tumor progression.

In PitNETs, CK18 plays a unique role as both a diagnostic and a biological marker. CK18 immunohistochemistry is integral to PitNET classification, particularly through the identification of fibrous bodies in sparsely granulated somatotroph tumors and Crooke’s cell changes in corticotroph tumors [20,21]. The observed association between CK18 negativity and invasive or hormonally inactive PitNETs supports the concept that loss of CK18 reflects cytoskeletal remodeling and reduced cellular differentiation, potentially contributing to more aggressive tumor behavior. Collectively, these findings suggest that CK18 may serve as an important integrative biomarker linking structural differentiation, functional activity, and invasive potential in PitNETs, warranting further investigation in combination with molecular, electrophysiological, and imaging-based markers [46].

As MRI is the gold standard for diagnosis, radiomics, consisting of the conversion of images into mineable data and subsequent analysis for decision support, has been gaining attention in recent years [47]. Preoperative assessment of pituitary macroadenoma consistency is critical for surgical planning and minimizing recurrence rates. However, the correlation between T2-weighted MRI signal intensity and tumor hardness remains controversial. While some studies link T2-hypointensity to increased hardness [48,49,50,51], others find no significant correlation [52,53,54,55], likely because T2 signals are confounded by factors like collagen density, intratumoral hematoma, or calcification [56].

Previous studies have demonstrated associations between T2-weighted MRI findings and tumor consistency, proliferative activity, secretory status, and response to somatostatin analogues in selected pituitary tumor subtypes [57,58,59,60]. In the present study, however, the imaging analysis was limited to conventional morphological MRI findings, and neither quantitative T2-weighted signal intensity nor functional measures of intracellular calcium signaling or endoplasmic reticulum organization were assessed. Consequently, the present data do not permit mechanistic conclusions regarding a relationship between RyR2 expression and MRI phenotype.

CK18-negative PitNETs exhibited significantly higher RyR2 expression than CK18-positive tumors. This finding represents an association between two immunohistochemical variables and does not demonstrate altered intracellular calcium signaling, endoplasmic reticulum organization, secretory activity, cellular excitability, or a causal relationship between CK18 and RyR2. The biological basis of this association, if any, requires investigation in dedicated functional studies. Because CK18 staining patterns are related to pituitary tumor differentiation and histopathological subtype, the observed association between CK18 status and RyR2 expression may partly reflect differences in tumor subtype composition. The present cohort was too small to reliably stratify the CK18–RyR2 relationship across individual histopathological lineages or subtypes. Although RyR2 expression did not differ significantly according to hormonal activity, larger studies with detailed lineage-based stratification are required to determine whether the observed CK18–RyR2 association is independent of tumor subtype.

Accordingly, these results should be considered preliminary and hypothesis-generating. Larger independent cohorts are required to confirm the RyR2–CK18 association, while dedicated functional studies would be necessary to determine whether any biological relationship exists between these markers.

## 4. Materials and Methods

### 4.1. Subjects and Ethical Approval

The study was conducted in accordance with the Declaration of Helsinki and was approved by the Kaunas Regional Biomedical Research Ethics Committee, Lithuanian University of Health Sciences, Kaunas, Lithuania (Approval No. BE-2-47; 14 December 2016). This exploratory observational study used prospective patient recruitment in the Ophthalmology and Neurosurgery Departments of the Lithuanian University of Health Sciences Hospital from January 2017 to January 2026. All participants provided written informed consent before inclusion. MRI data were subsequently reviewed retrospectively by an experienced radiologist.

Although recruitment covered an extended period, the present analysis represents a selected single-center tissue-based cohort rather than an epidemiological series of all patients diagnosed with PitNET during this period.

**Figure 1:**
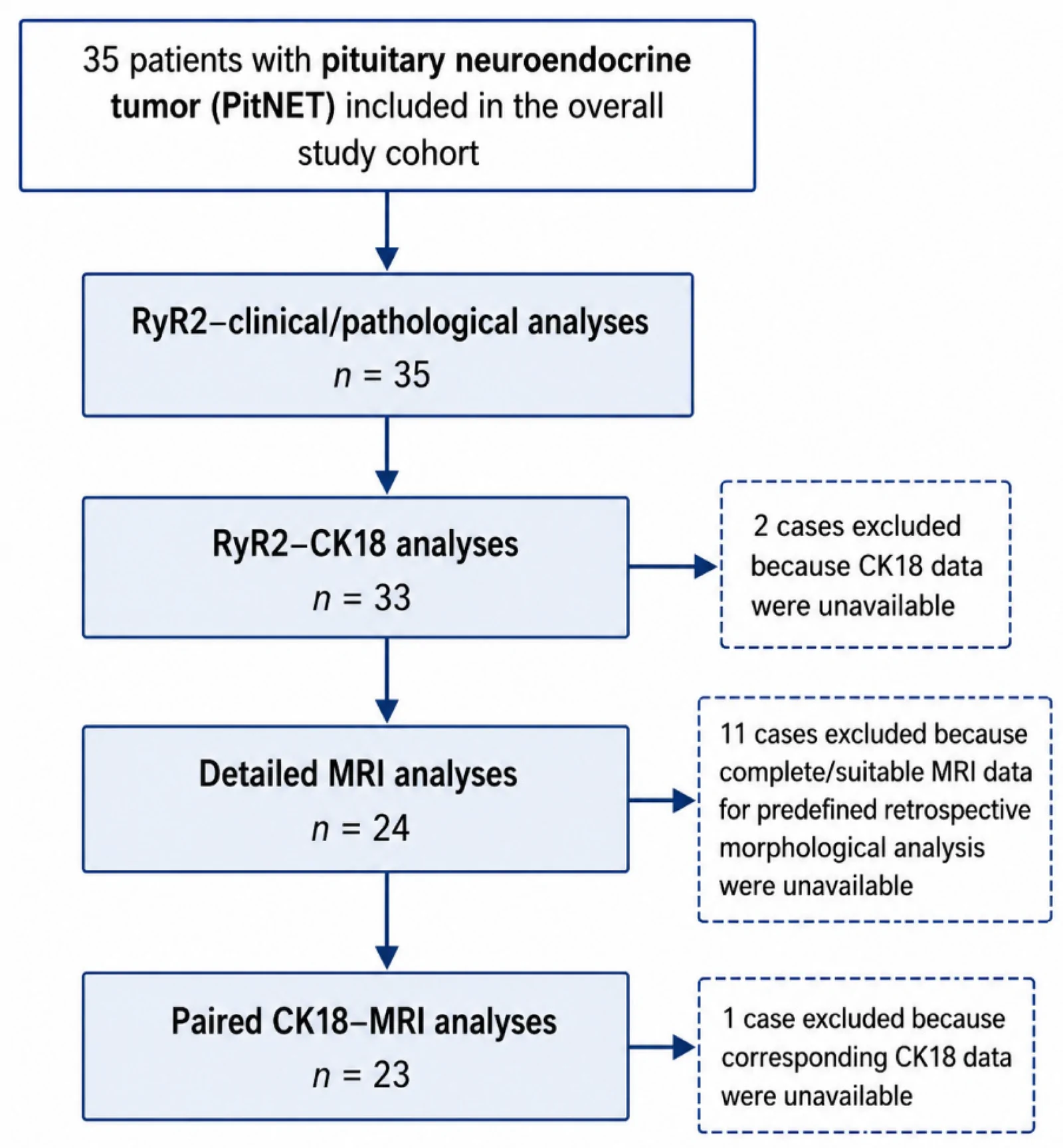
Flow diagram of patient inclusion in the study analyses. A total of 35 patients with pituitary neuroendocrine tumor were included in the overall cohort. Thirty-five patients were included in RyR2–clinical/pathological analyses, 33 in RyR2–CK18 analyses, 24 in detailed MRI analyses, and 23 in paired CK18–MRI analyses.

The study included 35 eligible cases of PitNETs enrolled according to predefined inclusion and exclusion criteria [61]. Complete MRI data suitable for the predefined detailed morphological analyses were available for 24 patients. The remaining 11 patients were not included in the detailed MRI analysis because complete preoperative MRI datasets were unavailable in the institutional archive and/or did not contain all sequences required for the predefined assessment. Paired CK18-MRI data were available for 23 patients, as one MRI-evaluable case lacked corresponding CK18 data. The flow of patients through the different study analyses is summarized in Figure 1.

### 4.2. Invasiveness Evaluation

For the detailed retrospective MRI analysis, the 24 PitNETs with available imaging data were evaluated using MRI findings, following our previously published methodology [62]. Suprasellar extension, parasellar extension, and sellar floor involvement were categorized using the Wilson–Hardy classification (Hardy classification modified by Wilson) [63].

Suprasellar and parasellar extension were graded from stages A to E, while sellar floor erosion was graded from I to IV. Tumors with grade III (localized sellar destruction) or grade IV (diffuse destruction) erosion were considered invasive. Cavernous sinus involvement was assessed using the Knosp classification, where only grades 3 and 4 indicate true cavernous sinus invasion: grade 0 indicates no cavernous sinus involvement; grades 1 and 2 represent extension toward the cavernous sinus; grade 3 indicates lateral extension to the internal carotid artery within the cavernous sinus; and grade 4 indicates complete encasement of the intracavernous carotid artery. Knosp grades 3 and 4 were considered indicative of true cavernous sinus invasion. Based on preoperative MRI measurements, PitNETs were defined as macroadenomas when the maximal diameter was ≥10 mm and as microadenomas when <10 mm.

### 4.3. Brain Imaging

**Figure 2:**
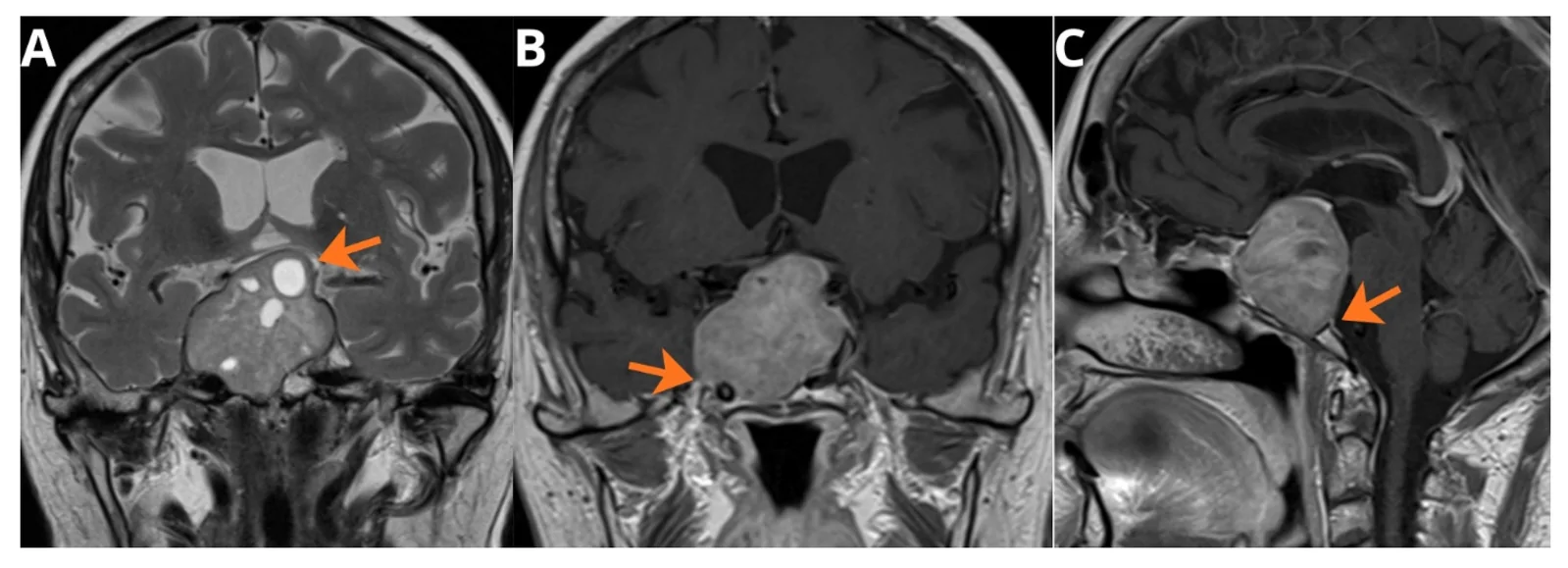
Representative MRI images of pituitary neuroendocrine tumor (PitNET). (**A**) Coronal T2-weighted image; (**B**) Coronal and (**C**) Sagittal contrast-enhanced T1-weighted MRI images. The image demonstrates a giant PitNET macroadenoma with heterogeneous enhancement, suprasellar extension with elevation and compression of the optic chiasm, bilateral cavernous sinus invasion with total or near-total encasement of the internal carotid arteries, and posterior/inferior extension toward the clivus and pontine cistern. The orange arrows indicate (**A**) a cystic component within the tumor, (**B**) cavernous sinus involvement with encasement of the internal carotid artery, and (**C**) posterior/inferior tumor extension toward the clivus and pontine cistern.

The 24 PitNETs with available MRI data were assessed using the predefined conventional imaging findings, in line with our previously reported methodology [62]. Preoperative imaging was performed on 1.5 T MRI systems (MAGNETOM Avanto; Siemens Healthcare GmbH, Erlangen, Germany) using a head coil and a standard pituitary imaging protocol. The protocol included pre-contrast T1-weighted sagittal and coronal sequences, pre-contrast T2-weighted turbo spin-echo (TSE) coronal images, and post-contrast T1-weighted coronal and sagittal images following gadodiamide administration (Omniscan; GE Healthcare AS, Oslo, Norway) (Figure 1). MRI data were retrospectively reviewed by an experienced radiologist. The radiologist was blinded to the immunohistochemical and relevant clinical data during the MRI assessment. Figure 2 shows MRI images of a pituitary macroadenoma with cystic degeneration: (A) a coronal T2-weighted image, (B) a coronal contrast-enhanced T1-weighted image, and (C) a sagittal contrast-enhanced T1-weighted image. The imaging analysis was limited to conventional qualitative assessments of tumor size, extension, and invasion; T2-weighted signal intensity was not quantified, and radiomic, texture, diffusion, or volumetric analyses were not performed.

### 4.4. Ryanodine Receptor 2 (RyR2) Immunohistological Evaluation

To evaluate the percentage of RyR2 in immunohistological tissue samples of PitNETs, we used QuPath v0.6.0, an open-source software for biological image analysis (Table 3). RyR2 expression was detected using a monoclonal antibody (clone EPR26288-70; Abcam Limited, Cambridge, UK; ab302716). This antibody was selected because it is a recombinant rabbit monoclonal antibody specific for RyR2 and is validated by the manufacturer for immunohistochemical analysis of formalin-fixed, paraffin-embedded human tissue. Manufacturer validation includes RyR2 immunoreactivity in human cardiac muscle and other tissues, as well as additional validation by Western blot and immunoprecipitation. Specific validation data for this antibody in human pituitary tumor tissue were not available. Immunohistochemical analysis was performed using the Roche Ventana BenchMark ULTRA PLUS (Ventana Medical System, Inc., Tucson, AZ, USA) in accordance with the manufacturer’s recommendations. Positive and negative tissue controls were included during the immunohistochemical staining procedure. Human myocardial tissue was used as the positive control, whereas human liver tissue was used as the negative control. The program was trained manually: using the Annotation function, the area (>5000 cells) was marked, and then all cells were differentiated (Cell detection). RyR2-positive cells, RyR2-negative cells, and artifacts were manually marked separately. The trained program automatically analyzed the images using the Object Classifier function in QuPath v0.6.0. In separate images of digital immunohistochemical tissue samples, after manually selecting an area covering at least 5000 cells, the program counted the total number of RyR2-negative and RyR2-positive cells. The minimum threshold of 5000 cells was used as a standardized sampling criterion to ensure that percentage estimates were based on a sufficiently large cell population and to reduce variability associated with small regions of interest. The same minimum threshold was applied to all analyzed samples. Regions of interest were selected according to the same predefined morphological criteria, prioritizing well-preserved tumor tissue with clearly identifiable cells and minimal artifacts. ROI selection and RyR2 quantification were performed while blinded to CK18 status and clinical and MRI findings. The same trained object classifier and classification criteria were applied consistently to all specimens. The data were systematized, and the percentage of RyR2-positive cells was calculated using Microsoft Excel for Microsoft 365 (Microsoft Corporation, Redmond, WA, USA). The RyR2-positive cell percentage was calculated as: [number of RyR2-positive cells/(number of RyR2-positive cells + number of RyR2-negative cells)] × 100. Cells classified as artifacts were excluded from the calculation. Thus, the analyzed RyR2 variable represents the percentage (%) of RyR2-positive cells within the selected tumor tissue area.

### 4.5. Cytokeratin 18 (CK18) Immunohistological Evaluation

Immunohistochemical analysis was performed using the Dako Omnis automated staining system (Agilent Technologies, Inc., Santa Clara, CA, USA) in accordance with the manufacturer’s recommendations, as previously described in our research [41]. CK18 expression was detected using a monoclonal antibody (clone DC-10; Vitro S.A., Sevilla, Spain). Stained slides were digitized using a Pannoramic 250 FLASH III scanner (3DHISTECH Ltd., Budapest, Hungary) and evaluated by a qualified pathologist at the Clinic of Pathological Anatomy of the LUHS using 3DHISTECH SlideViewer 2.9.0 software (3DHISTECH Ltd., Budapest, Hungary), following the WHO Classification of Endocrine and Neuroendocrine Tumors. The pathologist was blinded to the MRI findings and relevant clinical information during the immunohistochemical assessment. CK18 immunoreactivity was categorized as positive or negative, with positive cases further classified according to staining pattern (diffuse or focal) and subcellular localization (cytoplasmic, perinuclear, fibrous body, or ring-like perinuclear) (Table 4).

### 4.6. Statistical Analysis

Statistical analyses were performed using IMB SPSS Statistics for Windows, version 31.0.0.0 (IBM Corp., Armonk, NY, USA). Data normality was evaluated with the Shapiro–Wilk and Kolmogorov–Smirnov tests. As most variables were not normally distributed, non-parametric methods were used. RyR2 percentage values are presented as medians with interquartile ranges (IQR), calculated as the difference between the 75th and 25th percentiles (Q3–Q1). Group differences were assessed using the Mann–Whitney U test, and *p* < 0.05 was considered statistically significant. Associations between CK18 status and categorical MRI findings were assessed using Fisher’s exact test because of the small subgroup sizes and low expected cell counts. For Mann–Whitney U comparisons, effect sizes were calculated as r = |Z|/√N, where Z represents the standardized Mann–Whitney test statistic and N the total number of observations included in the comparison. Effect sizes of approximately 0.10, 0.30, and 0.50 were interpreted as small, moderate, and large, respectively. Because this was an exploratory pilot study that included all eligible cases available during the recruitment period, no formal a priori sample-size calculation was performed; the analyses were considered hypothesis-generating. No adjustment for multiple comparisons was applied because the analyses were exploratory and hypothesis-generating; therefore, all reported *p*-values are nominal and should be interpreted cautiously.

## 5. Conclusions

In this cohort of 35 PitNET patients, RyR2 expression showed no statistically significant associations with core clinical features (tumor size, invasiveness, hormonal activity, recurrence, or gender) or the evaluated conventional MRI findings. The only statistically significant finding was higher RyR2 expression in CK18-negative than in CK18-positive tumors. Given the exploratory design, limited sample size, and lack of functional validation, this association should be considered hypothesis-generating. Larger independent cohorts are required to confirm these findings and determine their biological relevance.

### Limitations

This study should be interpreted in light of several limitations. The main limitation is the relatively small cohort (*n* = 35), with MRI analyses available only for a subset of patients (*n* = 24), which reduces statistical power and may have limited the ability to detect small or moderate associations. Because matched healthy pituitary tissue cannot be ethically obtained through surgery, the study focused on comparisons between pituitary adenoma subgroups rather than tumor-versus-normal tissue comparisons. In addition, some MRI subgroups were very small, limiting reliable comparisons. Because detailed MRI analyses were restricted to patients with available and suitable imaging data, selection bias cannot be excluded. Patients with unavailable or incomplete MRI data may have differed from those included in the imaging subgroup, which may limit the generalizability of the MRI-related findings. Multiple exploratory comparisons were performed without formal adjustment for multiplicity, increasing the risk of type I error; therefore, statistically significant findings should be regarded as hypothesis-generating and require independent validation. The MRI analysis was restricted to conventional qualitative morphological characteristics; quantitative T2-weighted signal intensity, radiomic, texture, diffusion, and volumetric analyses were not performed. RyR2 assessment was based on immunohistochemical percentage estimates, which are semi-quantitative and may be influenced by intratumoral heterogeneity and technical variation. Although standardized ROI-selection criteria and the same trained QuPath classifier were applied to all specimens, no formal intra-observer or inter-observer reproducibility assessment was performed, which may represent an additional source of measurement variability. In addition, further representative RyR2 immunohistochemical images illustrating the range of staining patterns were not available for retrospective inclusion, which limits the visual assessment of staining heterogeneity and independent appraisal of the immunohistochemical findings. RyR2 expression was assessed by immunohistochemistry only and was not independently validated by qPCR or Western blot; therefore, concordance between RYR2 mRNA and protein expression cannot be assessed. Genetic characterization of RYR2 was not performed, and no functional in vitro experiments were conducted to investigate the biological mechanisms underlying the observed associations. The limited sample size also precluded reliable stratified analysis of the CK18–RyR2 association according to individual pituitary tumor lineages or histopathological subtypes; therefore, residual confounding by tumor subtype cannot be excluded. Finally, as an observational study, other potential confounders (e.g., tumor subtype differences or prior treatments) could not be fully controlled. Future multicenter studies with prespecified sample-size calculations and larger, well-stratified cohorts will be necessary to validate these findings. Future studies should also investigate RyR2 together with broader molecular and histopathological biomarker panels to determine whether combined marker profiles provide greater biological or clinical information than RyR2 or CK18 alone.

## Figures and Tables

**Table 1:** Association between CK18 status and RyR2 percentage values.

Characteristic	RyR2 Percent Median (IQR)	*p*-Value
CK18 negative	2.49% (18.36%)	**0.006**
CK18 positive	0.06% (0.39%)	

Values are presented as median (IQR) percentages of RyR2-positive cells. Group differences were assessed using the Mann–Whitney U test.

**Table 2:** Association between MRI findings, RyR2 and CK18 in PitNET patients.

Characteristic		Number of Patients (%)	RyR2 Percentage (Median (IQR))	*p*-Value	CK18 Status, *n* (%)		*p*-Value
					Negative	Positive	
Suprasellar extension	Yes	20 (83.3%)	0.065 (1.04)	0.737	6 (85.7)	13 (81.2)	1.000
	No	4 (16.7%)	0.26 (0.91)		1 (14.3)	3 (18.8)	
Parasellar extension	Yes	22 (91.7%)	0.075 (1.13)	0.725	7 (100)	14 (87.5)	1.000
	No	2 (8.3%)	0.13 (-)		0 (0)	2 (12.5)	
Parasellar and intracranial extension	(0/1) minimal extension	11 (45.8%)	0.22 (0.45)	0.955	2 (28.6)	8 (50)	0.405
	(2/3) significant extension	13 (54.2%)	0.06 (1.43)		5 (71.4)	8 (50)	
Paracavernous/cavernous sinus extension grades (Knosp classification)	(0/1/2) non-invasive	11 (45.8%)	0.22 (1.09)	0.459	2 (28.6)	8 (50)	0.405
	(3/4) invasive	13 (54.2%)	0.06 (1.43)		5 (71.4)	8 (50)	
Inferior parasellar extension	(0/1/2) non-invasive	10 (41.7%)	0.125 (4.89)	0.371	4 (57.1)	6 (37.5)	0.650
	(3/4) invasive	14 (58.3%)	0.055 (1.13)		3 (42.9)	10 (62.5)	

RyR2 values are presented as median (IQR) percentages of RyR2-positive cells. RyR2–MRI comparisons were assessed using the Mann–Whitney U test. Associations between CK18 status and categorical MRI findings were assessed using Fisher’s exact test (two-sided). RyR2–MRI analyses were performed in 24 patients; paired CK18–MRI analyses were performed in 23 patients.

**Table 3:** RyR2 immunohistological evaluation.

First stage—selecting the area for analysis. After importing the digital image of the immunohistochemical slide into QuPath, the region where the cells are most clearly visible is marked (no overlap and few artifacts). An area containing at least 5000 cells is selected.	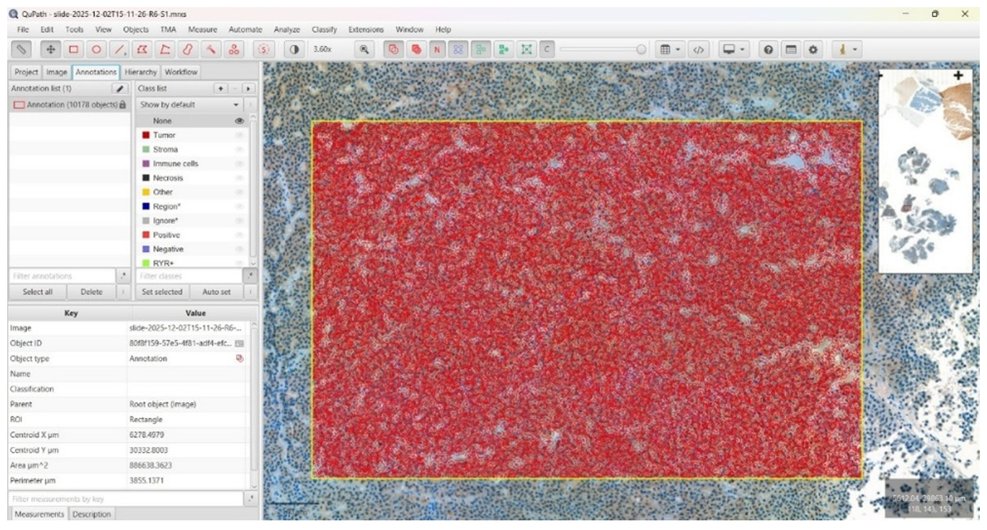
Second stage—cell detection and training the software. In the selected area, the program automatically delineates cell boundaries and preliminarily determines which cells are positive/negative. To obtain higher-quality results, the software is trained during the first analysis of images. When training the Object classifier, RyR2-positive and RyR2-negative cells are manually selected, and artifacts are annotated.	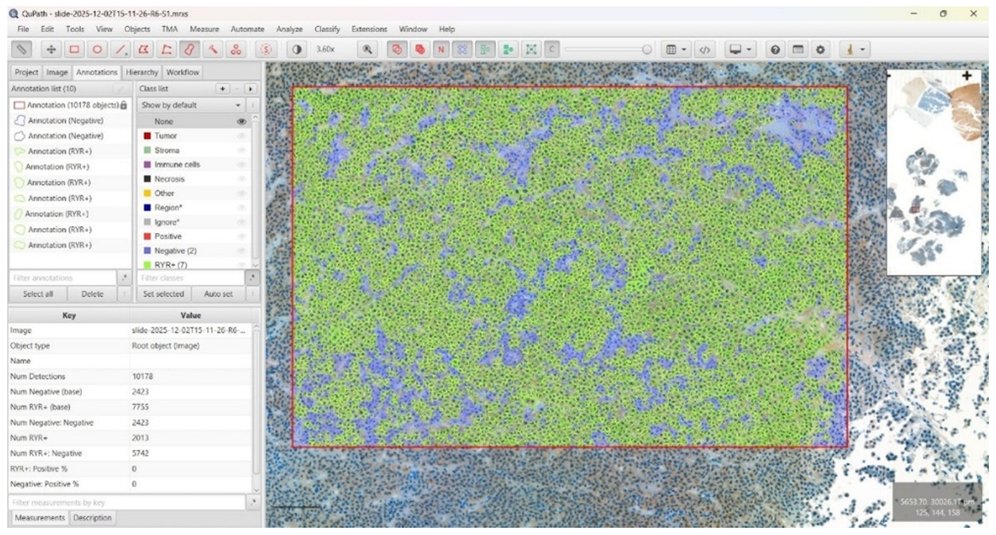
Third stage—cell counting. The trained program automatically counts, within the selected area, the number of RyR2-positive cells (Num RyR2+), RyR2-negative cells, and artifacts (Num Negative: Negative).	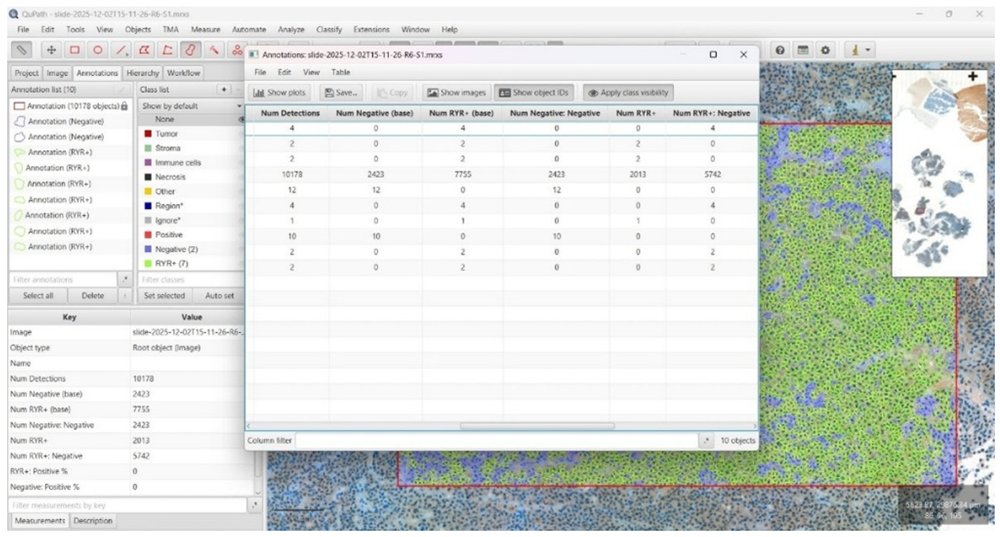

**Table 4:** Types of immunohistochemical reactions of PitNET with CK18 [41].

Tumor Type	Perinuclear Reaction
PIT1 lineage tumors: Somatotrophic PitNET Mammosomatotrophic PitNET etc. Representative RyR2 immunohistochemical staining with DAB + hematoxylin. Brown DAB staining indicates RyR2-positive immunoreactivity with a perinuclear staining pattern, whereas blue hematoxylin counterstaining identifies cell nuclei.	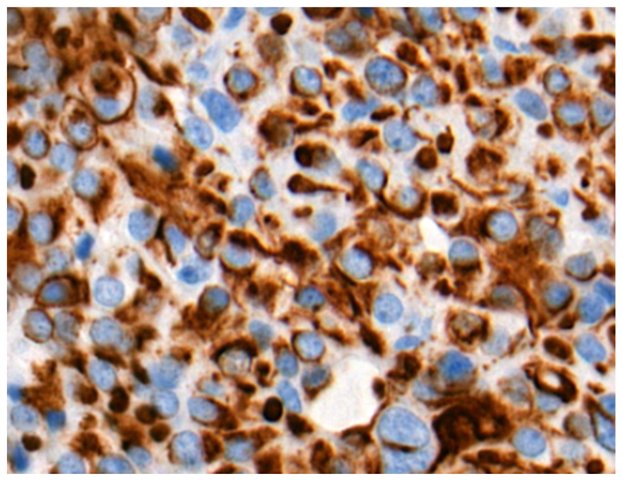
Tumor type	Ring-like perinuclear reactions
TPIT lineage tumors: Corticotrophic PitNET (Crooke adenoma) Representative RyR2 immunohistochemical staining with DAB + hematoxylin showing a ring-like perinuclear staining pattern. Brown DAB staining indicates RyR2-positive immunoreactivity surrounding the nuclei, whereas blue hematoxylin counterstaining identifies cell nuclei.	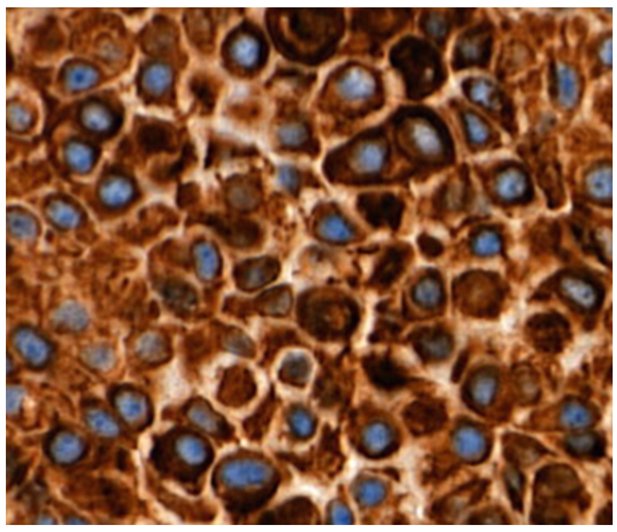
Tumor type	Fibrous body reactions
PIT1 lineage tumors: Somatotrophic PitNET Acidophilic stem cell PitNET etc. Representative CK18 immunohistochemical staining with DAB + hematoxylin showing a fibrous body staining pattern. Brown DAB-positive compact cytoplasmic aggregates represent CK18-positive fibrous bodies, whereas blue hematoxylin counterstaining identifies cell nuclei.	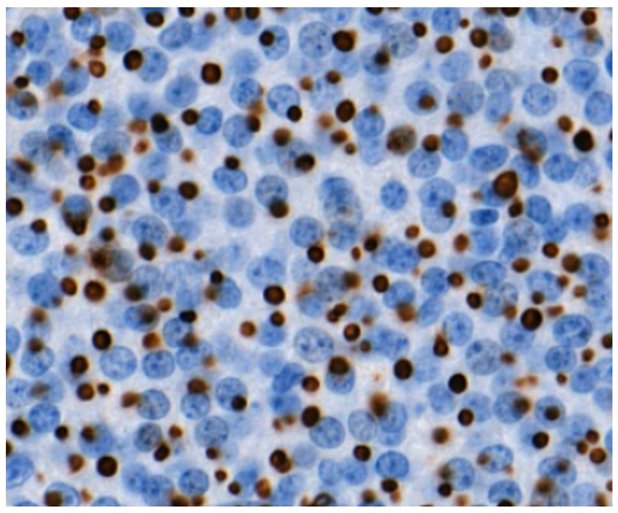

## Data Availability

The datasets used and/or analyzed during the current study are available from the corresponding author on reasonable request.
